# A Rare Case of Complicated Emphysematous Pyelonephritis

**DOI:** 10.7759/cureus.33941

**Published:** 2023-01-18

**Authors:** Solabomi O Ojeniyi, Sai Phani Sree Cherukuri, Olubunmi M Akharume, Philip Kanemo

**Affiliations:** 1 Internal Medicine, MedStar Union Memorial Hospital, Baltimore, USA; 2 Internal Medicine, Rapides Regional Medical Center, Alexandria, USA

**Keywords:** diabetes, nephrostomy tube, ct abdomen and pelvis with iv contrast, nephrectomy, emphysematous pyelonephritis

## Abstract

Emphysematous pyelonephritis is an acute severe necrotizing infection of the renal parenchyma and its surrounding tissues that results in the presence of gas in the renal parenchyma, collecting system, or perinephric tissue. The management of emphysematous pyelonephritis mainly depends on the extent of the disease.

In this report, we present the case of a 48-year-old male who presented with left flank pain and imaging findings of left-sided emphysematous pyelonephritis with extensions of air into the pararenal space as well as a 5.6 cm bladder stone and severe right-sided hydroureteronephrosis. He initially received bilateral nephrostomy tubes, a left-sided perinephric draining tube, and intravenous antibiotics; however, his symptoms persisted. Ultimately, the patient underwent open cystolitholapaxy and left nephrectomy, with eventual resolution of symptoms.

## Introduction

Emphysematous pyelonephritis (EPN) is an acute, potentially life-threatening, necrotizing infection affecting the renal parenchyma, collecting system as well as the surrounding tissue with the hallmark of the presence of gas within these structures. Common risk factors for EPN are diabetes mellitus (DM), obstructive uropathy, and immunosuppression [1]. Most reported cases of EPN are seen in patients with diabetes. This infection can be life-threatening if not adequately managed, and it has been reported to have a mortality rate as high as 80% [2,3].

*Escherichia coli* and *Klebsiella pneumoniae* are the most common organisms reported to be associated with EPN [4]. Management of EPN requires a multidisciplinary approach which includes medical management such as hydration, electrolyte management, broad-spectrum antibiotics, and strict glycemic control. This can be followed by decompressive techniques to relieve urinary obstruction. The goal of treatment is to preserve the affected kidney as possible, but in rare cases, an emergency nephrectomy may be required [5,6].

Here, we present an interesting case of a newly diagnosed diabetic patient found to have emphysematous pyelonephritis requiring open cystolitholapaxy and left nephrectomy.

## Case presentation

A 48-year-old male with a history of nephrolithiasis status post-stent placement about 20 years ago, bipolar disorder, and asthma presented to the emergency department with complaints of left flank pain for three days following a recent fall. He developed left-sided flank pain after a recent fall three days prior to the presentation. Left-sided flank pain was gradual in onset, dull in nature, later became 10 out of 10 in severity, with no known relieving factors, and was aggravated by a change of minimal exertion. When symptoms persisted, he presented to the emergency department. At presentation, on physical examination, the patient was tachycardic with left costovertebral angle tenderness. His initial blood test showed leukocytosis, elevated creatinine, hyperglycemia, and elevated hemoglobin A1C (Table 1).

**Table 1:** Significant laboratory results on presentation.

Blood test results (units)	Patient value	Reference range
White blood cells (/µL)	23,200	4,000–10,800
Creatinine (mg/dL)	1.6	0.6–1.0
Glucose (mg/dL)	273	65–140
Hemoglobin A1C (%)	7.1	5.7–6.4

Urinalysis showed glucosuria, proteinuria, hematuria, positive leukocyte esterase, bacteriuria, and elevated white blood cell count. The patient denied any previous history of DM.

**Figure 1:**
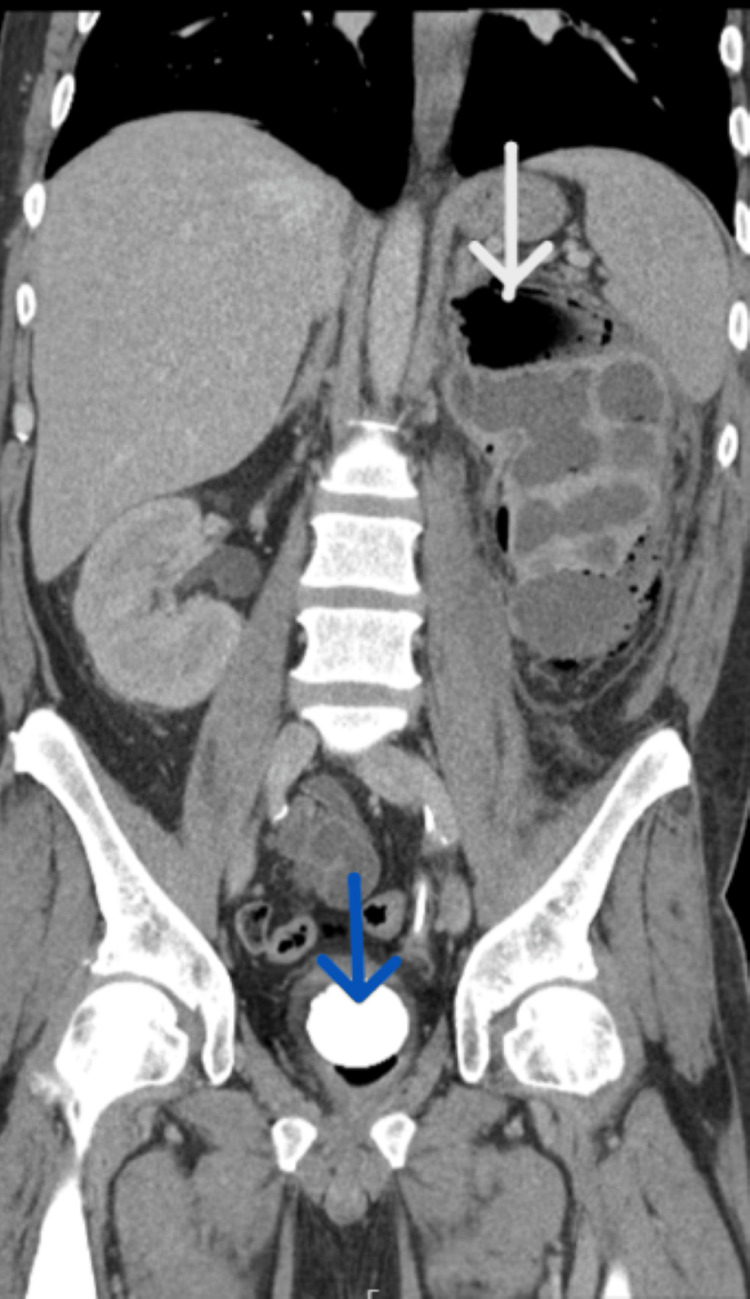
Coronal view of the CT scan shows an enlarged left kidney with areas of fluid collection (white arrow) and a 5.6 cm bladder stone (blue arrow).

CT of the abdomen and pelvis with intravenous (IV) contrast showed left-sided emphysematous pyelonephritis with extensions of air into the pararenal space, a 5.6 cm calcific density in the bladder, thought to be a bladder stone, and severe right-sided hydroureteronephrosis (Figure 1).

**Figure 2:**
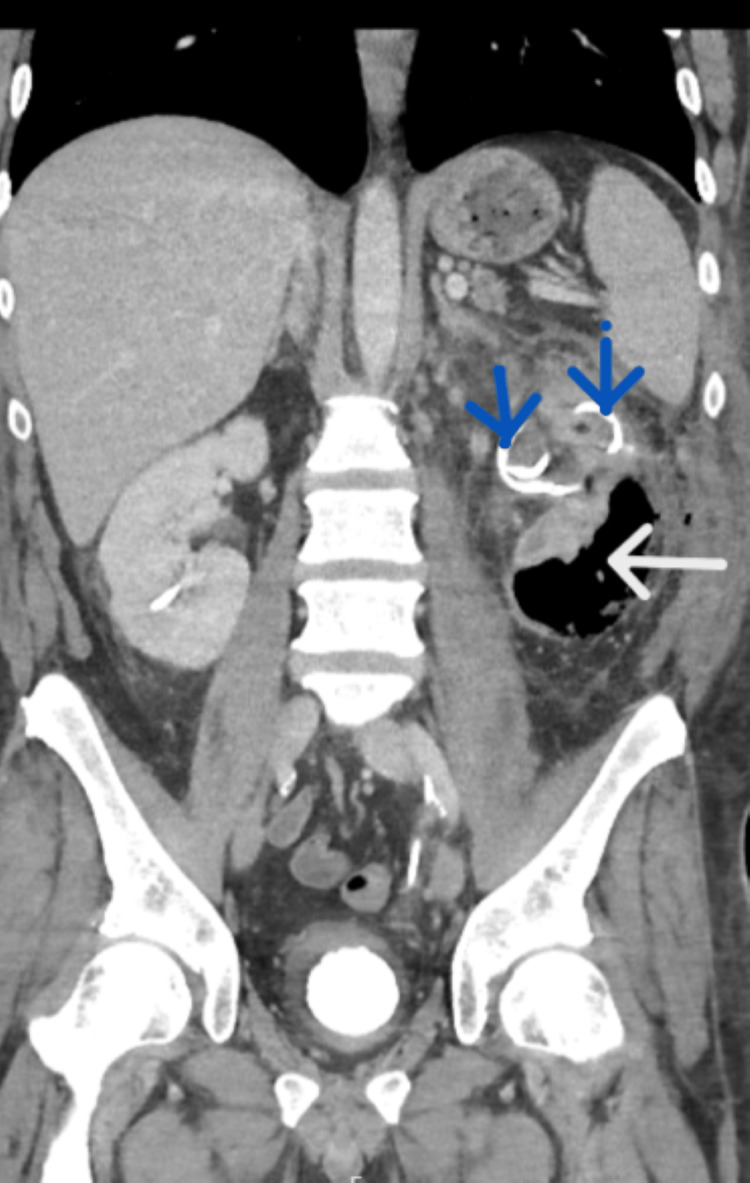
On day three of admission, a repeat coronal view of the CT scan shows the double-J ureteral stent and nephrostomy tube (blue arrows) with gas and undrained fluid in the left kidney (white arrow).

Based on physical examination, initial blood work, and CT findings, a diagnosis of EPN was made. He was admitted to the intermediate care unit. The interventional radiology team immediately inserted bilateral nephrostomy tubes along with a left-sided perinephric draining tube, which drained foul-smelling bloody fluid. He was started on aggressive IV fluids, broad-spectrum antibiotics (vancomycin and meropenem), an insulin regimen, and pain control. An initial decision by the multidisciplinary team (urology, infectious disease, internal medicine, and interventional radiology) was to continue with conservative management as the patient remained hemodynamically stable. Blood cultures grew *Enterococcus* species in four out of four bottles, and the patient was switched to IV ampicillin/sulbactam based on sensitivity results. On day three of admission, the patient continued to endorse severe left costovertebral angle tenderness. Repeat CT of the abdomen and pelvis revealed undrained fluid in the left kidney (Figure 2). Another left-sided perinephric tube was inserted.

Given persistent symptoms and findings on repeat CT of the abdomen and pelvis showing considerable undrained fluid with a very large bladder stone, the decision was made to perform a left nephrectomy with an open cystolitholapaxy. On day five of admission, the patient underwent an open cystolitholapaxy and a left nephrectomy. At the time of discharge, symptoms and acute kidney injury had resolved. The patient was discharged home on oral amoxicillin and clavulanic acid and was advised to follow up as an outpatient with urology and his primary care physician.

## Discussion

EPN is an uncommon suppurative infection of the kidney. It is characterized by the presence of gas in the renal system which might extend to the perinephric tissue [7]. Reported risk factors include DM and urinary tract obstruction, as seen in this case. There are very few reports of EPN in renal transplant recipients [8]. EPN is also reported to be more common in women compared to men.

The pathogenesis of pyelonephritis is poorly understood. Because this infection is commonly associated with diabetic patients, elevated blood glucose is a good environment for gas-forming organisms. This does not completely explain the pathologic and clinical manifestations of EPN [4,9].

*Escherichia coli* or *Klebsiella pneumoniae* are the two most common organisms associated with EPN, accounting for 65-100% of the isolates described in various retrospective reports. Other reported causative organisms include *Proteus*, *Enterococcus*, *Pseudomonas*, *Clostridium*, and, rarely, *Candida* species and *Aspergillus*. Some infections are polymicrobial [9-14].

Most patients complain of fevers, chills, flank pain or abdominal pain, nausea, and vomiting. The onset of symptoms may be abrupt or evolve slowly over two to three weeks. Costovertebral angle tenderness is considered the most common physical finding. This was the only finding on physical examination for our patient. 

EPN can be complicated by acute kidney injury and septic shock. Acute anuric kidney injury is an uncommon complication of EPN that can be seen in patients with bilateral infection or unilateral disease in a solitary functioning kidney. Rarely, a perirenal extension can progress to pneumomediastinum [15].

Imaging studies such as abdominal X-ray and ultrasonography have a very limited role in the diagnosis of EPN. CT scan is the preferred imaging modality for the diagnosis of EPN as it is more sensitive than other modalities [7]. It helps to demonstrate the presence and extent of gas. CT scan also helps to provide the prognosis of the disease. The extent of the disease on a CT scan correlates with the patient’s clinical outcome. A higher class, that is, a more extensive disease, has been associated with an increased risk of poor outcomes [5]. Table 2 describes the stages of the extent of EPN. The patient described in this case report falls into class 3B with the extension of air to the pararenal space.

**Table 2:** Class of disease defined on the basis of CT scan. A more detailed staging has been proposed by Huang and Tseng [7].

Class	Description
Class 1	Gas in the collecting system only
Class 2	Gas in the renal parenchyma without extension to the extrarenal space
Class 3A	Extension of gas or abscess to the perinephric space
Class 3B	Extension of gas or abscess to the pararenal space
Class 4	Bilateral emphysematous pyelonephritis (EPN) or solitary kidney with EPN

Initial medical management of EPN consists of intravascular fluids, antibiotics, and control of blood sugars. Percutaneous drainage is required to relieve obstruction and drain purulent material. However, nephrectomy is required for patients who have no improvement or show clinical deterioration despite optimized antibiotic therapy and catheter drainage.

## Conclusions

Approach to management of EPN depends on the extent of the disease based on CT scan findings. For patients found to have extensive disease from class 3A to class 4, a more aggressive mode of care must be involved to avoid adverse clinical outcomes. In this case, the patient underwent a left nephrectomy as a salvage procedure due to persistent symptoms despite the insertion of a nephrostomy tube to relieve the urinary obstruction.
